# Spectroscopic Investigation of the Interaction Between a Spermine-Functionalized Porphyrin and TERRA G-Quadruplexes

**DOI:** 10.3390/ijms27083424

**Published:** 2026-04-10

**Authors:** Gabriele Travagliante, Massimiliano Gaeta, Giorgio Campanella, Liliya A. Yatsunyk, Alessandro D’Urso

**Affiliations:** 1 Dipartimento di Scienze Chimiche, Università Degli Studi di Catania, Viale Andrea Doria, 6, 95125 Catania, Italy; travagliantegab@gmail.com (G.T.);; 2 Department of Chemistry and Biochemistry, Swarthmore College, 500 College Ave, Swarthmore, PA 19081, USA

**Keywords:** supramolecular interactions, TERRA, porphyrin and nucleic acid interactions, spectroscopy, topology modulation, antiparallel TERRA, G-quadruplex, ZnTCPPSpm4, circular dichroism

## Abstract

G-quadruplexes (G4s) are noncanonical nucleic acid structures involved in gene regulation and genome stability. Among them, the telomeric repeat-containing RNA (TERRA) forms biologically relevant RNA G4s (rG4s) that participate in telomere maintenance and genome stability. Although many ligands targeting DNA G4s have been reported, the recognition and modulation of RNA G4 topologies remain less explored. In this work, we investigated the interaction between TERRA and the spermine-functionalized Zn(II) porphyrin, ZnTCPPSpm4, using UV–vis absorption, fluorescence, resonance light scattering (RLS), and circular dichroism (CD) spectroscopy. In K^+^, where TERRA adopts a parallel G4 conformation, ZnTCPPSpm4 binds through a stepwise mechanism involving external end-stacking, forming discrete supramolecular complexes without altering the native topology. In contrast, under Na^+^ conditions, ZnTCPPSpm4 induces a gradual conformational rearrangement of TERRA from the antiparallel to a parallel-like G4 topology. A CD melting study showed that ZnTCPPSpm4 stabilizes the parallel RNA G4, while slightly destabilizing the antiparallel topology. Overall, our results demonstrate that ZnTCPPSpm4 is not a simple G4 binder, but a topology-selective ligand capable of remodeling TERRA G4 structures, highlighting the potential of metalloporphyrins as RNA G4-targeting scaffolds.

## 1. Introduction

**Figure 1:**
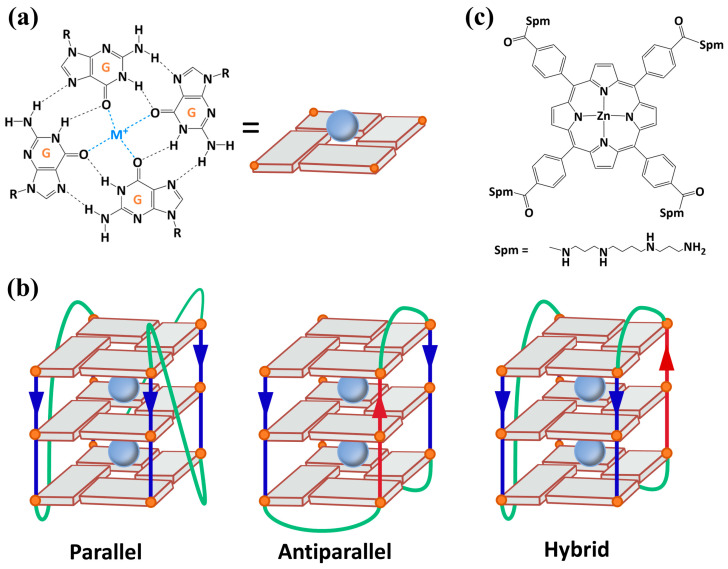
(**a**) Representation of a G-quartet formed by four guanine bases held together by Hoogsteen hydrogen bonds and stabilized by a central monovalent cation. (**b**) Schematic illustration of different G4 topologies, where guanines are depicted as cuboids with orange circles, straight lines represent the phosphate backbone, and green lines indicate the loop regions. Blue and red color of straight lines differentiates up and down orientation of RNA strand. (**c**) Chemical structure of ZnTCPPSpm4 porphyrin.

Nucleic acids, usually known for the Watson–Crick double helix, are now recognized for adopting a wide range of noncanonical secondary structures that play critical roles in several biological processes [1]. Among these, the G-quadruplex (G4), formed by guanine-rich sequences, is one of the most studied structures. Its structure consists of two or more planar guanine tetrads (G-quartet), composed of four guanine bases held together by Hoogsteen hydrogen bonds (Figure 1a), stacked on top of each other. Furthermore, G4 formation depends on the presence of monovalent cations, such as Na^+^ or K^+^, which intercalate between G-quartets and stabilize the negatively charged core of G4s interacting with oxygen atoms [2,3]. While DNA G4s can adopt parallel, antiparallel, and hybrid topologies (Figure 1b), RNA G4s mostly adopt a parallel topology, in which all four strands are oriented in the same direction [4]. This preference is principally ascribed to the presence of the 2′-OH group on the ribose sugar, which favors a specific sugar pucker and *anti* glycosidic conformation. Moreover, the 2′-OH group in RNA increases G4 stability by enabling intramolecular hydrogen-bonding interactions with nearby backbone oxygens or guanine base atoms, which reduce solvent exposure, restrict conformational flexibility, and favor a more compact RNA G4 architecture compared to DNA [4,5,6].

Although G4s were initially identified in DNA, notably in telomeres and oncogene promoter regions, a growing number of studies have highlighted the functional importance of RNA G4s (rG4s) in the transcriptome and in various diseases [7,8]. In particular, it has been demonstrated that mammalian telomeric DNA is transcribed into long non-coding RNAs (TERRAs), which are composed of repeating UUAGGG units capable of folding into G4 structures [9]. These rG4 structures have been confirmed in vivo using specific probes and in-cell NMR [10,11] and may protect TERRA from RNase degradation [12,13,14]; growing evidence suggests that rG4s form in vivo, particularly under cellular stress conditions [15]. Functionally, TERRA is essential for various telomere maintenance pathways, including telomerase activity regulation, chromatin structure modulation, and homologous recombination [16,17,18,19,20]. TERRA G4s specifically interact with crucial telomeric proteins, such as TRF2, and affect telomere stability and length [21,22]. The dysregulation of TERRA expression or altered rG4 structures is increasingly associated with telomere dysfunction and various pathologies, including neurodegenerative disorders and cancer [23,24,25,26]. Given their critical roles and specific interactions, TERRA G4s are being explored as promising therapeutic targets, with several G4 ligands demonstrating anti-proliferative effects in cancer models [27,28].

Within this expanding landscape of G4-targeting small molecules, porphyrins stand out as ideal, extensively characterized macrocyclic ligands. Recognized as one of the earliest classes of DNA ligands, their interactions with G4 DNA were first reported in 1998 [29] and recently also with G4 RNA [30,31,32,33]. Thanks to their planar aromatic system, porphyrins efficiently interact with G-quartets via π-π stacking and, if cationic, with the anionic phosphate backbone via electrostatic interactions. Generally, porphyrins primarily interact with G4s through end-stacking. Although intercalation is possible, it is generally less favorable for G4s formed by fewer than four G-tetrads [34,35,36,37,38,39,40]. Additionally, they can interact with the loops and grooves of G4s [41,42].

In this work, we investigated the interaction between the 22-nucleotide TERRA sequence (5′-AGGGUUAGGGUUAGGGUUAGGG-3′) and the achiral Zn(II)-meso-tetrakis(4-carboxyphenylspermine) porphyrin (ZnTCPPSpm4) (Figure 1c) using several spectroscopic techniques. We selected this porphyrin because under physiological conditions its four spermine side arms provide multiple positive charges [43], enhancing electrostatic interactions toward the negatively charged RNA backbone. Furthermore, these spermine moieties are also known to improve the biocompatibility of porphyrins, as polyamine analogs are efficiently taken up by cells through the polyamine transport system, especially in tumor tissues [44]. Moreover, previous studies have shown that ZnTCPPSpm4 can stabilize and recognize DNA G4 [45]. In addition, the presence of the Zn(II) center is not merely structural but confers specific coordination capabilities: Zn(II) can engage in axial coordination with nucleobase nitrogen atoms, particularly guanine N7, as previously demonstrated for Z-DNA, where ZnTCPPSpm4 acted as a selective probe and stabilizing agent [46], making it an excellent candidate for probing TERRA G4 folding and stability. Furthermore, the achiral nature of ZnTCPPSpm4 is a key advantage in this context, since it allows us to monitor the induced circular dichroism (iCD) signal that could appear only upon binding to a chiral scaffold such as a G4 [47]. iCD provides direct information on how the porphyrin interacts and arranges itself on the RNA structure.

The TERRA sequence used in this study has been previously characterized and is known to predominantly adopt a parallel rG4 structure [48,49,50,51,52]. However, recent studies have also reported the formation of an antiparallel topology under certain conditions [53,54]. This dual behavior makes it an ideal system for investigating how ZnTCPPSpm4 can distinguish between, and possibly modulate, different rG4 topologies.

The aim of this work was to characterize the binding of ZnTCPPSpm4 to TERRA and to assess whether this ligand can distinguish between, and potentially influence, different rG4 topologies. We therefore hypothesize that ZnTCPPSpm4 combines π-π stacking with multivalent electrostatic interactions and specific axial coordination of the Zn(II) center (to nucleobase sites) and acts as a topology-selective modulator of the rG4 structure.

## 2. Results

### 2.1. Effect of K^+^ and Na^+^ on the Topology and Stability of TERRA G-Quadruplexes

**Figure 2:**
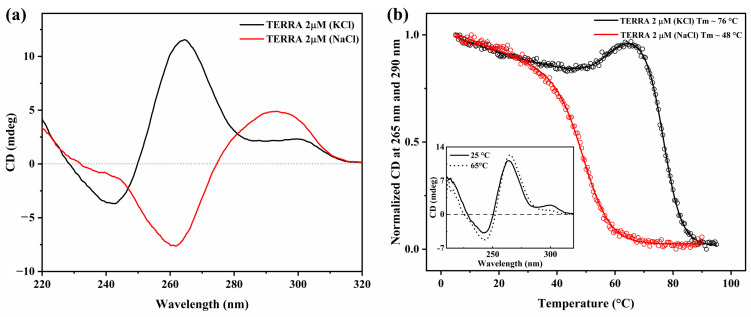
(**a**) CD spectra of TERRA (2 µM) in 10 mM Tris buffer (pH 7.2) supplemented with 100 mM KCl (black line) and 100 mM NaCl (red line). (**b**) CD melting curves of TERRA (2 µM) normalized to the [0, 1] range, recorded at 265 nm in KCl (black line) and at 290 nm in NaCl (red line). The inset shows the CD spectra of TERRA at 25 °C (solid black line) and at 65 °C (dashed black line) in Tris buffer supplemented with KCl.

The structural features and stability of the TERRA sequence were investigated in 10 mM Tris buffer (pH 7.2) in the presence of either 100 mM KCl or 100 mM NaCl by using UV-Vis (Figure S1) and circular dichroism (CD) spectroscopies (Figure 2). The CD spectrum of TERRA in NaCl solution (Figure 2a, red line) is characterized by a positive band around 290 nm and a negative band near 260 nm, which are typical of antiparallel G4 structures. Meanwhile, in the presence of KCl (Figure 2a, black line), TERRA displays a positive band at ~260 nm and a negative band near 240 nm, consistent with a parallel topology. A small positive band around 300 nm is also observed, a feature sometimes associated with the stacking of parallel G4s [55,56,57,58].

CD melting experiments were performed by monitoring the ellipticity at the characteristic wavelengths for each topology (Figure 2b). The antiparallel TERRA rG4 in NaCl (red line) displayed a single sigmoidal transition with a melting temperature (Tm) of approximately 48 °C. In contrast, the parallel rG4 in KCl (black line) showed a biphasic melting profile, with an initial structural perturbation around 30 °C followed by an unusual increase in CD intensity around 65 °C, immediately before the main melting transition at ~76 °C. This intermediate event may reflect the dissociation or rearrangement of weakly associated dimeric or oligomeric parallel G4 species into monomeric units upon increasing temperature. The inset of Figure 2b displays the CD spectra of TERRA in KCl at 25 °C and 65 °C recorded during the melting experiment, which reveal that the increased CD intensity is accompanied by the disappearance of the shoulder near 300 nm.

### 2.2. Interaction of ZnTCPPSpm4 with Parallel TERRA G-Quadruplex

#### 2.2.1. UV–Vis Absorption Studies

**Figure 3:**
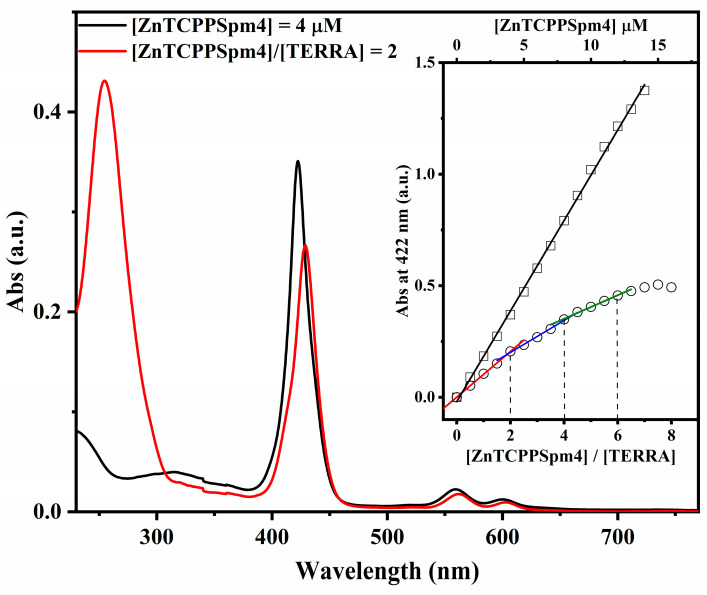
UV–vis absorption spectra of ZnTCPPSpm4 (4 µM) alone (black line) and in the presence of TERRA (2 µM, red line) in 10 mM Tris buffer (pH 7.2) supplemented with 100 mM KCl. The inset shows the break point analysis obtained by plotting the absorbance at 422 nm as a function of the [ZnTCPPSpm4]/[TERRA] ratio. The black line represents the linear trend of increasing the amount of ZnTCPPSpm4 alone in solution (open squares represent the experimental data points), while the differently colored segments indicate the distinct break points observed in the presence of TERRA (open circles represent the experimental data points).

The interaction between ZnTCPPSpm4 and TERRA rG4 was investigated using UV–vis absorption spectroscopy (Figure 3). The absorption spectrum of ZnTCPPSpm4 (4 µM, black line) displays a sharp Soret band centered at 422 nm. In the presence of TERRA (2 µM, red line), the Soret band exhibits hypochromicity (~24%) together with a red shift (∆λ = 7 nm).

To obtain quantitative insights into the interaction mechanism, UV–vis titrations were performed by adding increasing amounts of ZnTCPPSpm4 (up to 8 equiv.) to TERRA (2 µM) (Figure S2a). The absorbance at Soret maximum, 422 nm, was plotted as a function of the [ZnTCPPSpm4]/[TERRA] ratio (Figure 3, inset, circles) and of [ZnTCPPSpm4] alone in solution (Figure 3, inset, squares), respectively. The straight black line in the inset of Figure 3 shows that ZnTCPPSpm4 under these conditions does not change its aggregation state. Similarly, low-to-no aggregation was detected in water and in 5K buffer (Figure S3), consistent with our previous work [59]. In the presence of TERRA, the break-point analysis of titration data revealed three distinct break points at 2, 4, and 6 equivalents of porphyrin per TERRA (Figure 3, inset, squares). These break points indicate a stepwise binding process and the sequential formation of multiple supramolecular complexes characterized by different binding stoichiometries and distinct apparent molar extinction coefficients. We determined the apparent binding constant using the Peacocke–Skerrett method [60] to be K_app_ = 9.57 × 10^5^ M^−1^ (Figure S4). At higher porphyrin excess ([ZnTCPPSpm4/[TERRA] = 8:1), the absorbance decreased due to precipitation, likely associated with extensive porphyrin aggregation.

#### 2.2.2. Emission and Resonance Light Scattering Studies

**Figure 4:**
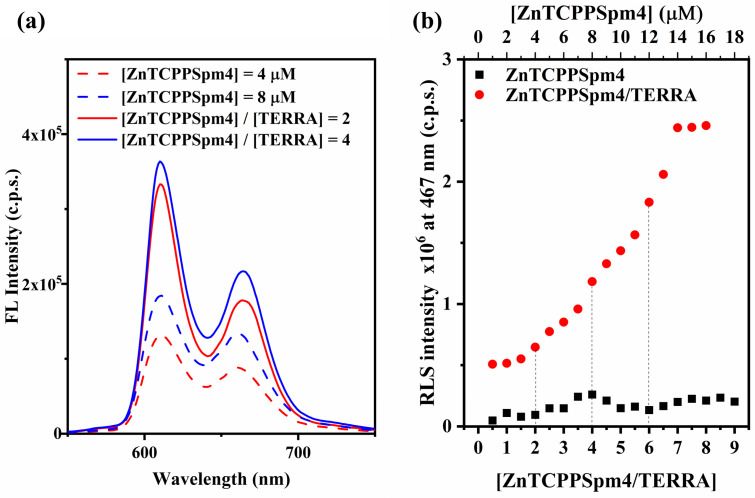
(**a**) Fluorescence emission spectra of ZnTCPPSpm4 alone at concentrations of 4 µM (red dashed line) and 8 µM (blue dashed line) and in the presence of TERRA (2 µM) at porphyrin/TERRA molar ratios of 2:1 (red solid line) and 4:1 (blue solid line). (**b**) Plot of the RLS intensity at 467 nm vs. the concentration of ZnTCPPSpm4 in the presence of TERRA (red circles) and alone (black squares). Vertical dashed lines indicate the [ZnTCPPSpm4]/[TERRA] ratios at which break points were observed in the UV–vis titration. All experiments were done in 10 mM Tris buffer (pH 7.2) supplemented with 100 mM KCl.

The emission spectra of ZnTCPPSpm4 were monitored in the absence and presence of TERRA (Figure 4a). ZnTCPPSpm4 alone (4 µM) displayed two emission bands centered at ~610 nm and ~665 nm (Figure 4a, red dashed line). In the presence of TERRA (2 µM), both emission bands increased in intensity, while the spectral profile remained unchanged (Figure 4a, red solid line). This trend became more evident upon the gradual addition of ZnTCPPSpm4 to a fixed TERRA concentration, where the emission intensity continued to increase up to a [ZnTCPPSpm4]/[TERRA] ratio of 4:1 (Figure 4a, blue line).

Resonance light scattering (RLS) measurements were performed to further characterize the interaction between ZnTCPPSpm4 and TERRA (Figure 4b and Figure S5). In the absence of TERRA, ZnTCPPSpm4 showed only a weak RLS signal across the investigated concentration range (1–16 µM), confirming the absence of intense aggregation of the porphyrin under these conditions (Figure S5a), in agreement with our earlier UV-vis dilution experiments (Figure S3). In contrast, when ZnTCPPSpm4 was titrated in the presence of TERRA (2 µM), a progressive increase in the RLS intensity was observed (Figure S5b), suggesting the formation of a well-organized ZnTCPPSpm4-TERRA rG4 complex. The trend is clearly reflected in Figure 4b, where the RLS intensity at 467 nm increases progressively with the [ZnTCPPSpm4]/[TERRA] ratio up to approximately 7:1, after which a plateau-like behavior is reached. Notably, the RLS intensities recorded at 2:1, 4:1, and 6:1 reflect the concentration regimes where break points were detected in the UV–vis titration, indicating changes in the supramolecular organization of the system.

#### 2.2.3. Circular Dichroism Studies

**Figure 5:**
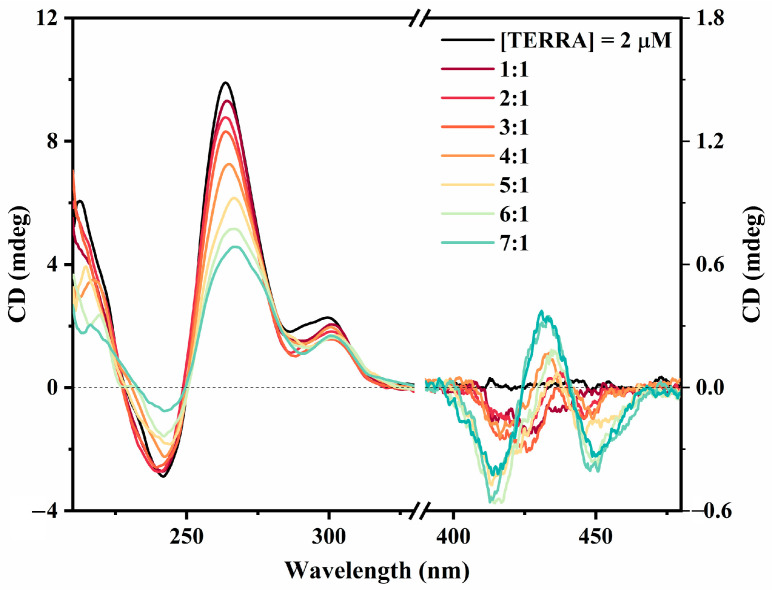
CD spectra of TERRA (2 µM) in 10 mM Tris buffer (pH 7.2) supplemented with 100 mM KCl in the absence (black line) and in the presence of increasing amounts of ZnTCPPSpm4. Spectra are reported at molar ratios [ZnTCPPSpm4]/[TERRA] ranging from 1:1 to 7:1, indicated by a progressive color scale.

We employed CD spectroscopy to investigate the structural integrity of the TERRA rG4 alone and in the presence of ZnTCPPSpm4. The complete CD titration is reported in Figure 5. The CD spectrum of TERRA alone (Figure 5, black line) is mostly unaltered in its features but decreases in intensity (in the 220–330 nm region) upon the progressive addition of ZnTCPPSpm4. This observation confirms that rG4’s secondary structure is maintained upon complex formation. Notably, a weak induced CD (iCD) signal appears in the Soret region of ZnTCPPSpm4 (400–460 nm), which increases with an increased porphyrin:TERRA ratio. This iCD profile displays a distinct trisignate shape, negative–positive–negative according to excitonic coupling theory, that indicates a strong communication among porphyrin chromophores interacting with a chiral scaffold [61].

### 2.3. Interaction of ZnTCPPSpm4 with Antiparallel TERRA Induces a Parallel rG4 Topology

**Figure 6:**
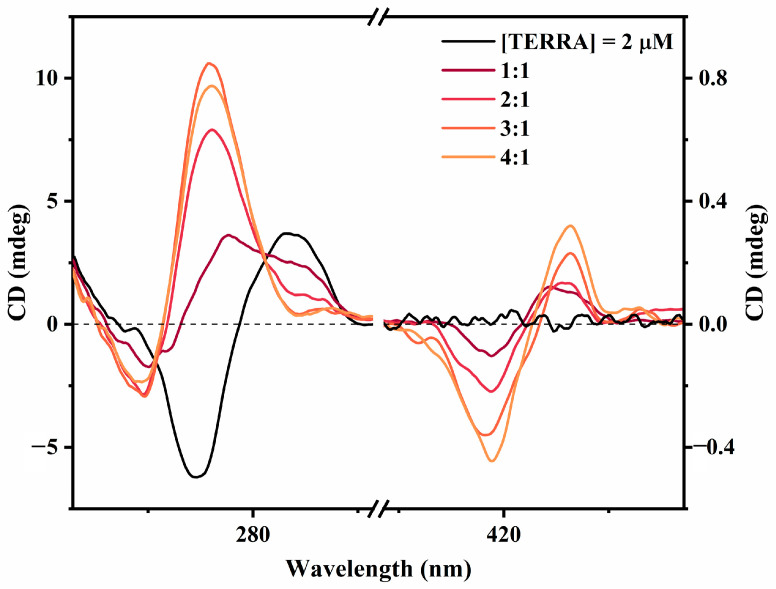
CD spectra of TERRA (2 µM) in 10 mM Tris buffer (pH 7.2) supplemented with 100 mM NaCl in the absence (black line) and in the presence of increasing amounts of ZnTCPPSpm4. Spectra are reported at molar ratios [ZnTCPPSpm4]/[TERRA] ranging from 1:1 to 4:1, indicated by a progressive color scale.

To investigate the effect of ZnTCPPSpm4 on the antiparallel form of TERRA, CD titration experiments were performed in 10 mM Tris buffer (pH 7.2) containing 100 mM NaCl. The CD spectrum of free TERRA (Figure 6, black line) undergoes significant spectral changes upon the addition of ZnTCPPSpm4. The characteristic 290 nm band progressively decreases in intensity and shifts toward 265 nm, while a new negative band emerges near 240 nm. These spectral modifications indicate a gradual transition from an antiparallel to a parallel topology of TERRA rG4.

Additionally, a positive bisignate iCD signal appears in the Soret region (400–460 nm), confirming the interaction between the achiral porphyrin and the chiral RNA scaffold. The iCD amplitude increases with porphyrin concentration, consistent with the progressive formation of porphyrin–RNA complexes.

### 2.4. ZnTCPPSpm4 Modulates TERRA G-Quadruplex Topology by Destabilizing the Antiparallel and Stabilizing the Parallel Fold

**Figure 7:**
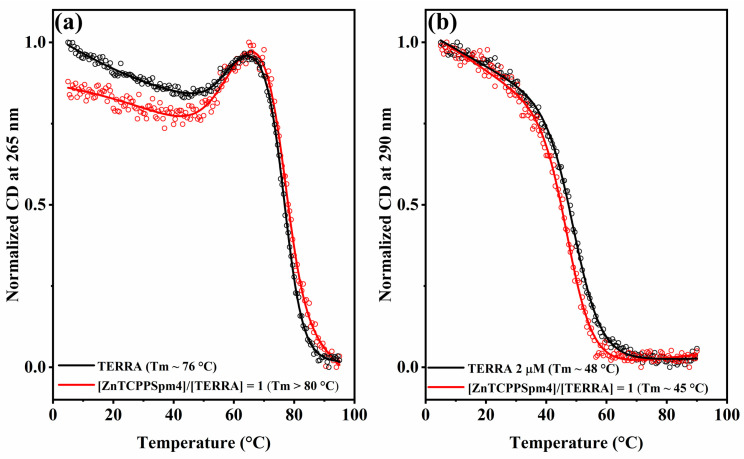
CD melting of TERRA (2 µM) in 10 mM Tris (pH 7.2) supplemented with (**a**) 100 mM KCl and (**b**) 100 mM NaCl, in the absence (black lines) and in the presence (red lines) of ZnTCPPSpm4 at a 1:1 ratio (2 µM). Signals were monitored at 265 nm (K^+^, parallel) and 290 nm (Na^+^, antiparallel) and normalized to the [0, 1] range.

Finally, to investigate how ZnTCPPSpm4 affects the stability of TERRA in the different topologies, CD melting experiments were performed. CD melting curves were recorded for TERRA in 10 mM Tris (pH 7.2) containing either 100 mM KCl (Figure 7a) or 100 mM NaCl (Figure 7b), in the absence (black) and presence (red) of ZnTCPPSpm4 at a 1:1 ratio. The signal was monitored at 265 nm in K^+^ and at 290 nm in Na^+^, and the results were normalized to the [0, 1] range. In KCl, the addition of ZnTCPPSpm4 shifts TERRA’s melting profile to a higher temperature (Tm > 80 °C) while maintaining a comparable sigmoidal shape (Figure 7a). In NaCl, the addition of ZnTCPPSpm4 shifts TERRA’s melting profile to a lower temperature (Tm ~ 45 °C) with no appreciable change in curve shape (Figure 7b).

### 2.5. Interaction of ZnTCPPSpm4 with Calf Thymus DNA

**Figure 8:**
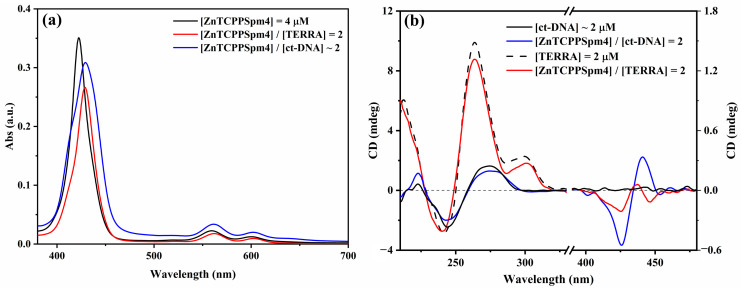
(**a**) UV–vis absorption spectra of ZnTCPPSpm4 (4 µM) alone (black line), in the presence of TERRA (2 µM) (red line), and in the presence of ct-DNA (2 µM) (blue line) recorded in 10 mM Tris buffer (pH 7.2) supplemented with 100 mM KCl. (**b**) CD spectra of ct-DNA (2 µM) (solid black line) and TERRA (2 µM) (dashed black line) in the same buffer conditions, and in the presence of ZnTCPPSpm4 at [ZnTCPPSpm4]/[ct-DNA] = 2 (blue line) and [ZnTCPPSpm4]/[TERRA] = 2 (red line).

To evaluate the selectivity of ZnTCPPSpm4 toward G4 structures, its interaction with calf thymus DNA (ct-DNA) was investigated as a representative B-DNA model. As shown in Figure 8a, the UV–vis spectrum of ZnTCPPSpm4 in the presence of ct-DNA displays a lower hypochromicity (Figure 8a, blue line) compared to that in the presence of TERRA (Figure 8a, red line) and a limited red shift in the Soret band, indicative of a non-specific external binding mode. Consistently, CD measurements (Figure 8b, blue line) reveal an induced bisignate signal in the porphyrin absorption region and no significant perturbation of the characteristic B-DNA CD profile, suggesting a disordered distribution of the porphyrin along the duplex.

The apparent binding constant obtained for ct-DNA (K_app_ = 2.16 × 10^5^ M^−1^) was calculated using the same UV–vis titration approach used for the TERRA sequence (Figure S6).

## 3. Discussion

Firstly, to explore the topology of the selected TERRA sequence under our experimental conditions, we examined its folding behavior and stability using UV-Vis (Figure S1), CD and CD melting experiments in the presence of two different monovalent cations (Figure 2). Our data confirm that the cation present in solution plays a crucial role in determining the topology adopted by the TERRA G4. In line with previous studies, Na^+^ ions favor the formation of antiparallel conformations, while K^+^ ions stabilize the parallel topology, which is typically dominant in rG4s [53,54]. The higher thermodynamic stabilization imparted on TERRA by K^+^ compared to Na^+^ (ΔT ~ 28 °C) is consistent with the optimal ionic radius of K^+^ for coordination within the central channel of the stacked G-quartet.

The CD melting curve in Na^+^ is monophasic, but in K^+^ it shows a biphasic profile (Figure 2b, black line): a first transition between ~30 and 65 °C, marked by a modest increase in ellipticity, and a second sharper melting event centered around 76 °C. A plausible explanation for this behavior is that TERRA adopts a dimeric rG4 formed by the stacking of two rG4 units, with the lower-temperature transition reflecting the disruption or rearrangement of this higher-order species into monomeric rG4 units. The subsequent higher-temperature transition would then correspond to the unfolding of the stable monomeric parallel rG4. Importantly, this interpretation does not interfere with any of the analyses or conclusions presented in this work (see Appendix A). In sum, K^+^ not only promotes the parallel topology of TERRA but also enhances the overall stability of the structure.

Secondly, to verify if and how ZnTCPPSpm4 interacts with the parallel TERRA rG4, several spectroscopic techniques were used, each providing complementary information on the binding mode, the supramolecular organization of the complexes, and the potential modulation of rG4 stability by this cationic porphyrin.

Regarding the UV–vis titration results (Figure 3), the hypochromism (~24%) and red shift in the Soret band (Δλ ~ 7 nm) observed at the ratio [porphyrin]/[TERRA] 2:1 reflect strong π-π stacking interactions between the aromatic porphyrin macrocycle and the G-quartet surface. The moderate hypochromic effect and limited red shift across the titration suggest an end-stacking mode rather than intercalation, in agreement with established criteria where Δλ ≤ 10 nm and hypochromicity < 35% are associated with terminal stacking [38,62,63,64]. The breakpoint analysis (Figure 3, inset) further confirms the sequential formation of distinct supramolecular species not in equilibrium with each other, as demonstrated by the change in slope compared with the linear behavior of ZnTCPPSpm4 alone (Figure 3, inset black line). The first breakpoint is detected at a 2:1 [ZnTCPPSpm4]/[TERRA] ratio and is associated with a marked increase in hypochromicity, reflecting a strong binding event. This stoichiometry is consistent with the end-stacking of two porphyrins on different or the same terminal G-quartets of TERRA. The increased hypochromicity suggests strong electronic communication between porphyrins and TERRA, potentially suggesting that ZnTCPPSpm4 binds to two ends of TERRA [61].

A second break point appears at a 4:1 ratio (Figure 3, inset, blue line), accompanied by a stronger hypochromicity and comparable red shift (Figure S2b). This data can be attributed to progressive porphyrin–porphyrin stacking interactions, where additional porphyrins bind to those already stacked at the G-quartets, giving rise to small porphyrin aggregates templated by the G4 structure. Such multistep formation of stacked assemblies has been widely reported for sperminated porphyrins interacting with G4 DNA [40].

A third breakpoint is observed at a 6:1 ratio (Figure 3, inset green line), accompanied by an increased hypochromicity (from ~24% to ~52%) and a slight additional red shift (from ∆λ = 7 nm to ∆λ = 10 nm) (Figure S2c); this behavior is indicative of increased porphyrin aggregation, likely driven by porphyrin–porphyrin stacking interactions at higher ligand concentrations. Beyond this point, the slope decreases and eventually the absorbance drops (8:1 ratio), indicating an aggregation and partial precipitation of the ZnTCPPSpm4-TERRA complex.

Overall, these observations suggest that two distinct interaction regimes can be identified. At low ligand-to-RNA ratios (up to approximately 2:1), the spectroscopic features are consistent with specific end-stacking interactions of ZnTCPPSpm4 at the terminal G-quartets of the G4 structure. At higher ligand ratios (≥4:1), the increasing hypochromicity and enhanced RLS intensity indicate the progressive formation of porphyrin–porphyrin stacked assemblies templated by the G-quadruplex scaffold, which at still higher ratios evolve into larger supramolecular aggregates.

From these titration data, the apparent binding constant obtained using the Peacocke–Skerrett method [60] was Kapp = 9.57 × 10^5^ M^−1^ (Figure S4). This value falls within the expected range for porphyrin–nucleic acid interactions: DNA G4 typically exhibits binding constants between 10^5^ M^−1^ and 10^7^ M^−1^ [35,45]; RNA rG4 exhibits constants between 10^5^ and 10^6^ M^−1^ [31,32,33]. For comparison, the apparent binding constant determined for ct-DNA (Kapp = 2.16 × 10^5^ M^−1^) is significantly lower than that measured for the TERRA G-quadruplex, indicating a weaker and less specific interaction with canonical duplex DNA. Thus, the affinity of ZnTCPPSpm4 for TERRA supports the formation of a stable yet typical rG4–porphyrin complex.

To further assess the G4-dependence of ZnTCPPSpm4 binding, additional experiments were performed in the presence of LiCl, a condition known to disfavor G-quadruplex formation. Under these conditions, TERRA displays a significantly reduced CD signal with a broad and poorly defined band (Figure S7a), indicating a largely unstructured or weakly folded conformation compared to the well-defined parallel G4 observed in KCl. Consistently, CD melting experiments (Figure S7b) reveal a markedly lower thermal stability (Tm ~ 55 °C). UV–vis titration analysis (Figure S7c,d) further shows that the apparent binding constant in LiCl is approximately 8 × 10^4^ M^−1^, one order of magnitude lower than that determined for TERRA in KCl, supporting a substantially weaker interaction in the absence of a stable G-quadruplex structure. Altogether, these results reinforce that the binding of ZnTCPPSpm4 is strongly dependent on the presence of a well-defined G4 topology.

Overall, these UV–vis findings demonstrate that ZnTCPPSpm4 binds TERRA rG4 through a stepwise process involving discrete supramolecular complexes and well-defined interactions, driven by π-π stacking and probably assisted by electrostatic interactions between the spermine arms and the RNA backbone, which is consistent with previous studies for DNA G4 [8,40,65] and RNA rG4 [31,32,33].

Fluorescence emission experiments further confirm the nature of ZnTCPPSpm4-TERRA complexes. In the presence of TERRA, the fluorescence intensity of ZnTCPPSpm4 is progressively enhanced up to a ~4:1 [ZnTCPPSpm4]/[TERRA] ratio (Figure 4a). This fluorescence “turn-on” can be rationalized by the accommodation of porphyrin molecules on the rG4 scaffold, where specific binding and ordered stacking disrupt porphyrin self-quenching, enforce rigidification, and shield the chromophores from solvent, thereby reducing non-radiative decay [40,63,66,67].

RLS experiments show that ZnTCPPSpm4 alone displays a negligible scattering intensity, whereas a higher intensity is observed upon porphyrin interaction with TERRA (Figure 4b and Figure S5). The immediate rise in RLS intensity reflects the formation of ordered supramolecular assemblies larger than the free porphyrin, already at low binding ratios. The RLS spectra show three regions of more pronounced intensity increase at [ZnTCPPSpm4]/[TERRA] ratios of approximately 2:1, 4:1 and 6:1 (Figure 4b), which correlate well with the breakpoints observed in the UV–vis titration. This correlation strongly supports the stepwise self-assembly mechanism detected through absorbance experiments. In the first regime (up to 2:1), the moderate increase in RLS intensity suggests the formation of compact ZnTCPPSpm4—TERRA complexes, consistent with end-stacking at the G-quartet surfaces. In the second and in the third regime (2:1 to 4:1 and 4:1 to 6:1), the increase in scattering intensity reflects the growth of porphyrin–porphyrin interactions templated by the rG4, in agreement with the excitonic interactions detected in fluorescence measurements. Beyond 6:1, the RLS signal rises sharply, indicating the formation of larger supramolecular assemblies, where porphyrin–porphyrin contacts dominate over porphyrin–G4 interactions. Finally, above a ratio of 7:1, the RLS intensity begins to level off, consistent with the onset of aggregation and the partial precipitation observed in UV–vis data.

Together, UV–vis, fluorescence and RLS results consistently indicate that ZnTCPPSpm4 does not bind TERRA as a simple 1:1 complex but engages in a hierarchical assembly process, transitioning from specific end-stacking interactions at low porphyrin loading to π-π stacked aggregates stabilized by the rG4 scaffold at intermediate ratios, and ultimately to self-associated supramolecular aggregates at high ligand excess.

CD spectroscopy offered additional information on how ZnTCPPSpm4 interacts with the parallel TERRA rG4 (Figure 5). The parallel signature of rG4 TERRA in K^+^ is preserved upon the addition of ZnTCPPSpm4 (up to a 6:1 [ZnTCPPSpm4]/[TERRA] ratio), showing that the porphyrin binds without a structural conversion of the parallel rG4. Nonetheless, the progressive decrease in ellipticity at 265 nm indicates that the porphyrin does influence the rG4 environment, likely by interacting at its external G-quartet surfaces.

In the Soret region of the CD spectrum (400–450 nm), where free achiral ZnTCPPSpm4 is CD-silent, a trisignate iCD profile becomes evident, featuring alternating positive and negative components. Such a multisegnate pattern suggests that ZnTCPPSpm4 molecules arrange in a chiral geometry around the rG4 scaffold, likely through ordered stacking interactions [40,68]. Although the iCD intensity remains low, its evolution from a 2:1 to a 4:1 ratio is accompanied by a slight shift and a small increase in intensity, consistent with increasing electronic communication between porphyrin chromophores. At 6:1 (Figure 5), the iCD amplitude increases and the signal becomes broader, suggesting the emergence of higher-order stacked aggregates with reduced conformational freedom. These observations align with the cooperative assembly detected using UV–vis breakpoints and RLS enhancement.

Having established the binding behavior of ZnTCPPSpm4 toward the parallel TERRA rG4, we next examined the interaction with the antiparallel form of TERRA to evaluate whether the ligand shows topology selectivity or induces structural rearrangement under Na^+^-stabilizing conditions. The CD titration results (Figure 6) clearly show that ZnTCPPSpm4 gradually promotes a conformational shift from antiparallel toward a parallel topology of TERRA. The appearance of a weak positive bisignate iCD signal in the Soret region and its gradually increasing amplitude suggests a partial ordering of the porphyrin units on the RNA scaffold, possibly through external stacking at the terminal G-quartets. ZnTCPPSpm4 appears to act as a molecular template that selectively stabilizes parallel G4 arrangements, thereby shifting the conformational equilibrium toward a parallel topology even under Na^+^-stabilizing conditions.

Consistent with these spectral changes, CD melting (Figure 7) corroborates a topology-dependent effect: in K^+^, monitoring at 265 nm, ZnTCPPSpm4 increases the melting temperature of TERRA (ΔTm ≥ +4 °C), whereas, in Na^+^, monitored at 290 nm, it produces a slight decrease (ΔTm ~ −3 °C). Thus, ZnTCPPSpm4 stabilizes the parallel fold of TERRA while marginally destabilizing the antiparallel one.

Analogous antiparallel-to-parallel G4 DNA conversions have been reported for several ligands [69,70,71,72], which share two key structural features: a planar aromatic core that stacks on the terminal G-quartet and cationic side chains that interact electrostatically with grooves or the central channel. These combined π-π and electrostatic interactions selectively stabilize the parallel topology, a mechanism consistent with the structural behavior observed for ZnTCPPSpm4 on TERRA.

## 4. Materials and Methods

### 4.1. Materials and Sample Preparation

Trizma base (Tris), hydrochloric acid (HCl), potassium chloride (KCl), sodium chloride (NaCl), lithium chloride (LiCl) and calf thymus DNA (ct-DNA) were purchased from Sigma-Aldrich (St. Louis, MO, USA)and used without further purification. A 10 mM Tris stock solution at pH 7.2 was prepared by dissolving the appropriate amount of Tris in 200 mL of ultrapure water (Purelab Flex system, Elga LabWater, Veolia, High Wycombe, UK; 18.2 MΩ·cm resistivity). The pH was adjusted to 7.2 with 1 M HCl. The buffer solution was divided into two aliquots: 100 mL were supplemented with KCl and the remaining 100 mL with NaCl to reach a final salt concentration of 100 mM.

The concentration for ct-DNA was determined spectroscopically through UV–vis using ε_260_ = 6600 L·mol^−1^·cm^−1^ per nucleotide.

The telomeric RNA (TERRA) sequence (5′-AGGGUUAGGGUUAGGGUUAGGG-3′) was purchased from Integrated DNA Technologies (IDT, Coralville, IA, USA) and used without further purification. The lyophilized oligonucleotide was dissolved in ultrapure water to obtain a stock solution of ~100 µM. The RNA concentration was determined through UV–vis spectroscopy at 90 °C using the extinction coefficient provided by IDT (ε_260_ nm = 236,900 L·mol^−1^·cm^−1^).

Sample solutions were prepared by diluting the TERRA stock in 10 mM Tris buffer (pH 7.2) to a final RNA concentration of 2 µM, either in Tris supplemented with 100 mM NaCl (to promote the antiparallel topology) or with 100 mM KCl (to promote the parallel topology). The annealing procedure was performed on the final sample solutions by heating at 95 °C for 5 min, followed by slow cooling to room temperature and storage at 4 °C overnight before use.

ZnTCPPSpm4 was synthesized according to the literature procedure [46]. Stock solutions were prepared by dissolving the solid in ultrapure water to obtain concentrations in the range of 2 × 10^−4^ M to 3 × 10^−4^ M. The concentration of the stock solutions was determined spectrophotometrically using ε_423_ = 133,766 M^−1^ cm^−1^ at pH 3.5 in aqueous HCl solution.

### 4.2. Electronic Circular Dichroism (ECD)

ECD spectra were recorded at 20 °C using a Jasco J-715 spectropolarimeter (JASCO, Tokyo, Japan) equipped with a single-position Peltier temperature control system. A quartz cuvette with a 1 cm path length was employed in all experiments. A solution of 2 µM TERRA was titrated with increasing amounts of ZnTCPPSpm4, and CD spectra were collected with the following parameters: scanning rate, 100 nm min^−1^; data pitch, 0.2 nm; digital integration time (D.I.T.), 4 s; bandwidth, 2.0 nm. Each spectrum was obtained as the average of at least three scans.

CD melting experiments were carried out at a porphyrin:TERRA ratio of 1:1, monitoring the ellipticity at 265 nm (parallel topology) and 290 nm (antiparallel topology) in the 5–90 °C temperature range, with a heating rate of 1 °C min^−1^ and a response time of 4 s. Melting profiles were analyzed using a multi-sigmoidal Boltzmann function:

$$y=y_{0}+\frac{A_{1}}{1+\exp\left(\frac{x-T_{m1}}{k_{1}}\right)}+\frac{A_{2}}{1+\exp\left(\frac{x-T_{m2}}{k_{2}}\right)}-\frac{A_{3}}{1+\exp\left(\frac{x-T_{m3}}{k_{3}}\right)}$$

where *y*_0_ represents the baseline, *A_i_* are the transition amplitudes, *T_mi_* are the apparent melting temperatures, and *k_i_* are the slope parameters related to cooperativity.

### 4.3. UV-Vis Absorption Spectroscopy

UV–vis spectra were recorded at 20 °C using a Jasco V-530 spectrophotometer (JASCO, Tokyo, Japan) with a 1 cm path-length quartz cell. The conditions were: scanning rate, 100 nm min^−1^; data pitch, 0.5 nm; bandwidth, 2 nm. Titrations of 2 µM TERRA were performed by adding increasing amounts of ZnTCPPSpm4.

Porphyrin–RNA titrations were analyzed by plotting the Soret band absorbance as a function of the [porphyrin]/[RNA] ratio. Breakpoints in the titration curves, identified by at least a 10% change in slope, indicated variations in complex stoichiometry [65,73]. Multiple straight-line segments correspond to the formation of distinct supramolecular species with specific extinction coefficients, while a single linear trend indicated either an equilibrium among all complexes or an unchanged aggregation state (as observed for porphyrin alone in buffer solution). Titrations were terminated when the slopes of the lines approached those observed for porphyrin in buffer alone or when precipitation occurs.

### 4.4. Fluorescence Spectroscopy and Resonance Light Scattering (RLS)

Fluorescence emission and RLS spectra were recorded at 20 °C using a Fluorolog FL-11 spectrofluorometer (Horiba Scientific, Kyoto, Japan) with a 1 cm path-length quartz cuvette. For fluorescence measurements, the following parameters were used: emission range, 550–800 nm; increment, 1.0 nm; averaging time, 0.1 s; one scan; excitation and emission slit widths, 2.5 nm. The excitation wavelength (λ_ex_ = 425 nm) was selected at the intersection point identified from the UV–vis titrations of ZnTCPPSpm4 alone and in the presence of TERRA, in order to minimize self-absorption effects.

## 5. Conclusions

In this work, we employed complementary spectroscopic techniques to investigate the interaction between the spermine-functionalized porphyrin ZnTCPPSpm4 and the telomeric RNA G-quadruplex (TERRA) adopting parallel (K^+^) and antiparallel (Na^+^) topologies. The results show that ZnTCPPSpm4 binds TERRA through a stepwise and topology-dependent mechanism governed by π-π stacking, multivalent electrostatic interactions, and possibly Zn(II) coordination.

Under K^+^ conditions, where TERRA adopts a parallel G4 topology, ZnTCPPSpm4 interacts mainly through external end-stacking at the terminal G-quartets, leading to the progressive formation of supramolecular assemblies templated by the G-quadruplex scaffold. UV–vis titration revealed distinct binding regimes, while fluorescence, RLS, and CD measurements consistently indicated the ordered growth of porphyrin aggregates without altering the parallel G4 structure. CD melting experiments further showed that ZnTCPPSpm4 slightly stabilizes the parallel topology.

In contrast, under Na^+^ conditions, the ligand promotes a gradual conformational rearrangement of TERRA from an antiparallel toward a parallel-like topology. CD titration showed the progressive loss of the antiparallel signature together with the emergence of spectral features characteristic of parallel G-quadruplexes, while CD melting indicated a modest destabilization of the original fold.

Overall, these findings indicate that ZnTCPPSpm4 behaves not only as a G-quadruplex binder, but also as a topology modulator capable of stabilizing parallel TERRA and promoting structural rearrangement of the antiparallel form. This work provides further insight into porphyrin–RNA G-quadruplex recognition and highlights spermine-functionalized metalloporphyrins as promising scaffolds for targeting RNA G-quadruplex structures.

## Data Availability

The original contributions presented in this study are included in the article/Supplementary Materials. Further inquiries can be directed to the corresponding author.

## Supplementary Materials

The following supporting information can be downloaded at: https://www.mdpi.com/article/10.3390/ijms27083424/s1.

## Abbreviations

The following abbreviations are used in this manuscript:

G4	G-quadruplex
rG4	RNA G-quadruplex
TERRA	Telomeric repeat-containing RNA
TRF2	Telomeric Repeat-binding Factor 2
ZnTCPPSpm4	Zinc(II)-meso-tetrakis(4-carboxyphenyl)spermine porphyrin
ct-DNA	Calf thymus DNA
iCD	Induced circular dichroism
CD	Circular dichroism
ECD	Electronic Circular Dichroism
RLS	Resonance light scattering
Tm	Melting temperature
D.I.T.	Digital integration time
λex	Excitation wavelength

## Appendix A

If TERRA adopts a dimeric parallel arrangement in KCl, the stoichiometric trends extracted from UV–vis break-point analysis would simply scale by a factor of two. Breakpoints observed at 2:1, 4:1, and 6:1 for the monomeric G4 would correspond to 1:1, 2:1, and 3:1 relative to a dimer, without altering any mechanistic interpretation. UV–vis signatures, fluorescence trends, RLS behavior, and CD responses all remain fully consistent with the binding model described for the monomeric G4. Therefore, the possible presence of a dimeric G4 does not affect any of the structural or mechanistic conclusions drawn for the interaction between ZnTCPPSpm4 and TERRA.
