# Supplementary material for: Spectroscopic Investigation of the Interaction Between a Spermine-Functionalized Porphyrin and TERRA G-Quadruplexes
Source: Int J Mol Sci. 2026 Apr 10;27(8):3424. doi: 10.3390/ijms27083424 (PMC13116396; doi:10.3390/ijms27083424)
Supplement: Supplementary file 1 [file ijms-27-03424-s001.zip › ijms-4194494-supplementary.pdf]

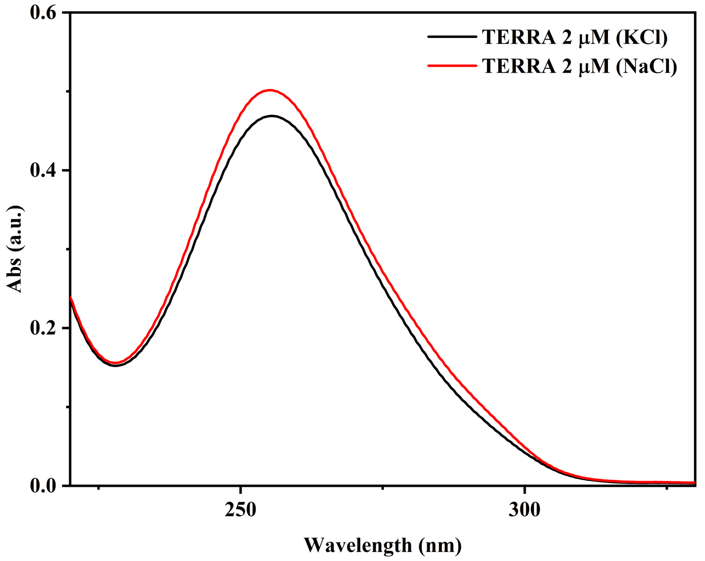

Figure S1. UV-vis spectra of TERRA (2 μM) in 10 mM Tris buffer (pH 7.2) supplemented with 100 mM KCl (black line) and 100 mM NaCl (red line).

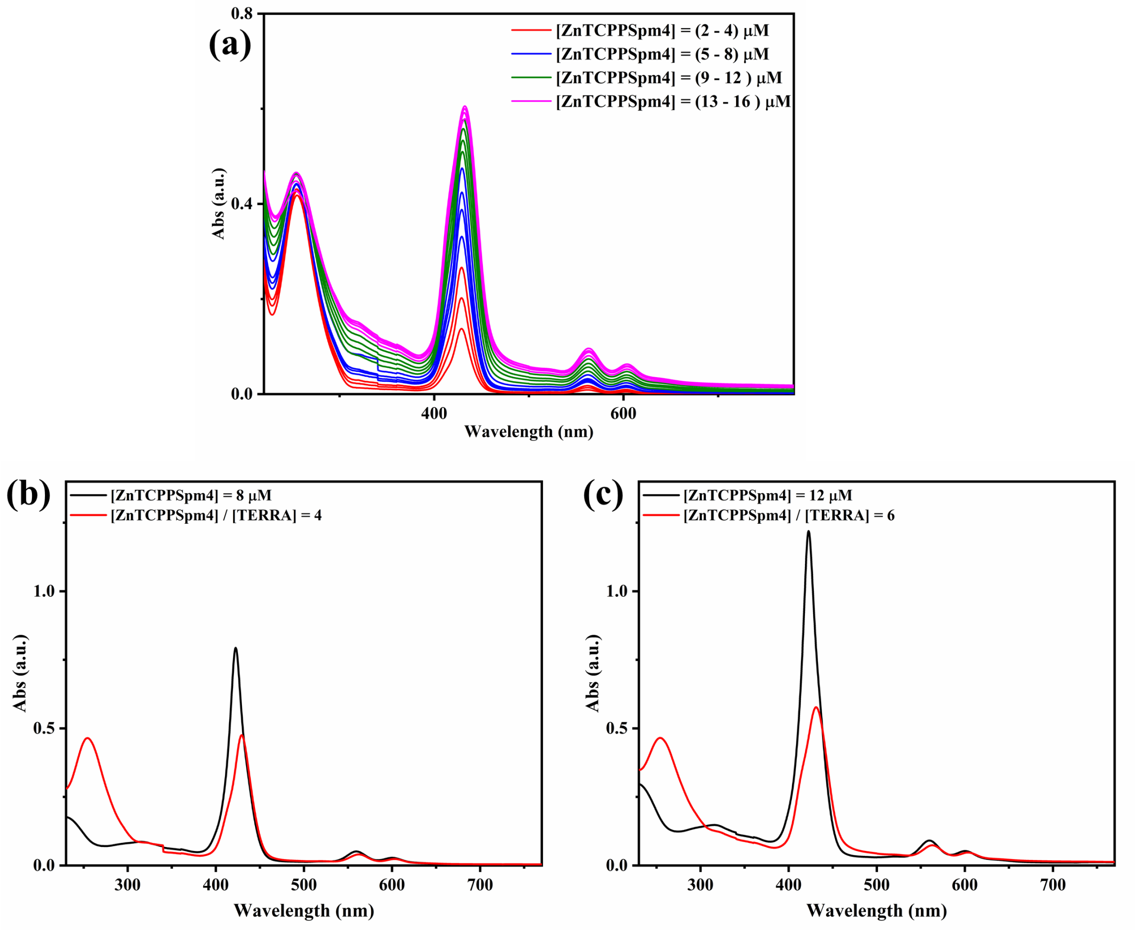

Figure S2. UV–vis titration of TERRA (2 μM) with increasing concentrations of ZnTCPPSpm4 (2–16 μM) in 10 mM Tris buffer (pH 7.2) supplemented with 100 mM KCl. ZnTCPPSpm4 was added in 1 μM increments. The color code is consistent with that used in the break-point plot of absorbance at 422 nm vs. the [ZnTCPPSpm$_4$]/[TERRA] ratio (inset of Figure 3). Panels **(b)** and **(c)** show the comparison between ZnTCPPSpm4 alone (black line) and in the presence of TERRA (red line) at porphyrin concentrations of 8 μM and 12 μM, respectively.

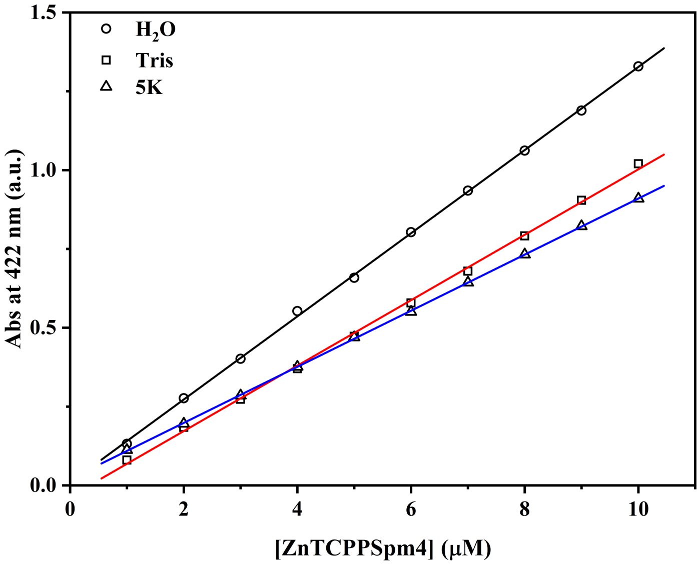

Figure S3. Variation in the absorbance of the ZnTCPPSpm4 Soret band (λmax = 422 nm) as a function of porphyrin concentration (μM) in: H_2O (circles), 10 mM Tris buffer (pH = 7.2) supplemented with 100 mM KCl (squares), and "5K buffer" consisting of 10 mM sodium cacodylate supplemented with 5 mM KCl and 95 mM LiCl (triangles).

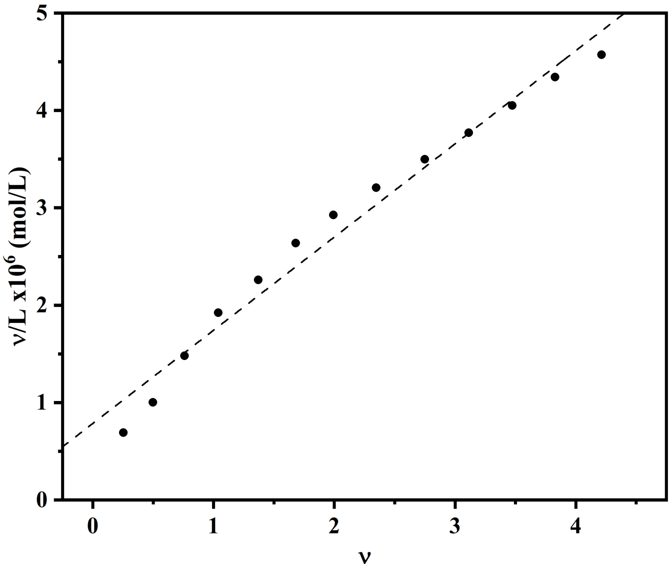

Figure S4. Scatchard plot for the interaction between ZnTCPPSpm4 and TERRA, calculated from the UV–vis titration. Titration data were analyzed, as previously reported in our recent work [65] using the Peacocke–Skerrett method [60] in order to calculate the values of ν (number of moles of bound porphyrin per mole of total RNA) and L (molar concentration of free porphyrin at equilibrium), which are required to construct the Scatchard plot (Figure S3). At each point of the titration, the fraction of bound porphyrin (α) was determined using the equation (Eq. 1):

$$\alpha = \frac{A_f - A}{A_f - A_b}$$

where:

A_f is the absorbance at the Soret maximum (422 nm) for the free porphyrin (ZnTCPPSpm4), A_b is the absorbance at the same wavelength under conditions of complete binding, achieved by adding TERRA at 20-fold excess over porphyrin concentration, A is the absorbance at any given point during the titration. The values of ν and L were plotted to generate the Scatchard plot (**Figure S4**), from which the apparent binding constant K_{app} was obtained as the absolute value of the slope in the linear region, following Eq. 1. The resulting K_{app} value is 9.57×10^5 M^{-1}.

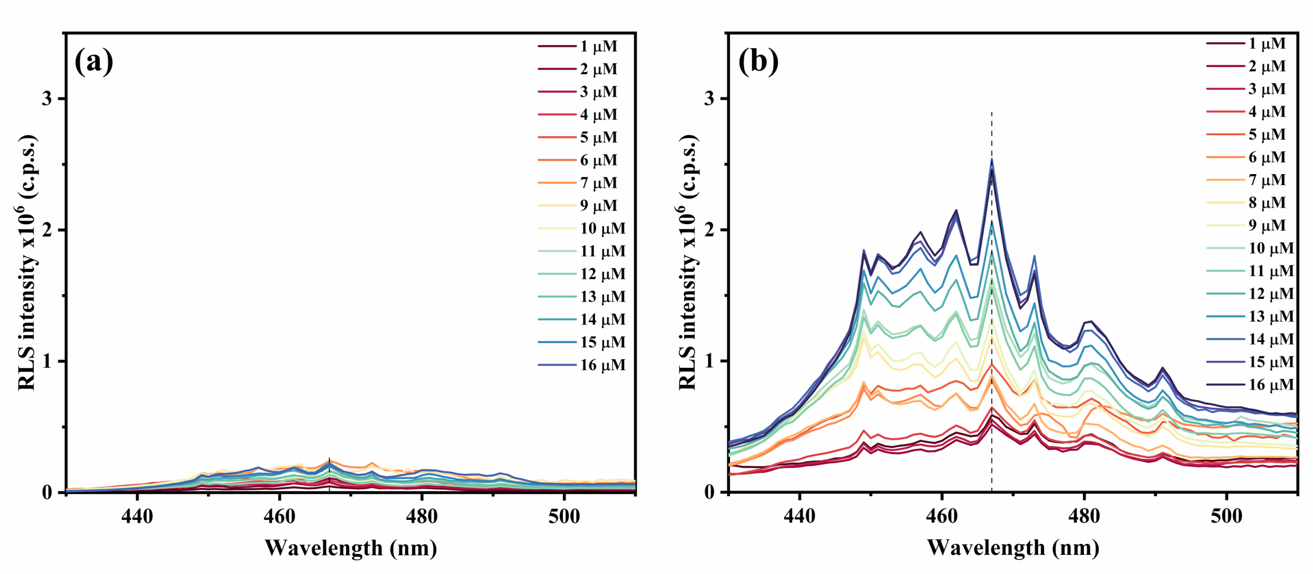

Figure S5. RLS spectra of ZnTCPPSpm4 in 10 mM Tris buffer (pH 7.2) supplemented with 100 mM KCl in the absence **(a)** and in the presence **(b)** of TERRA (2 µM). The porphyrin concentration was progressively increased from 1 to 16 µM.

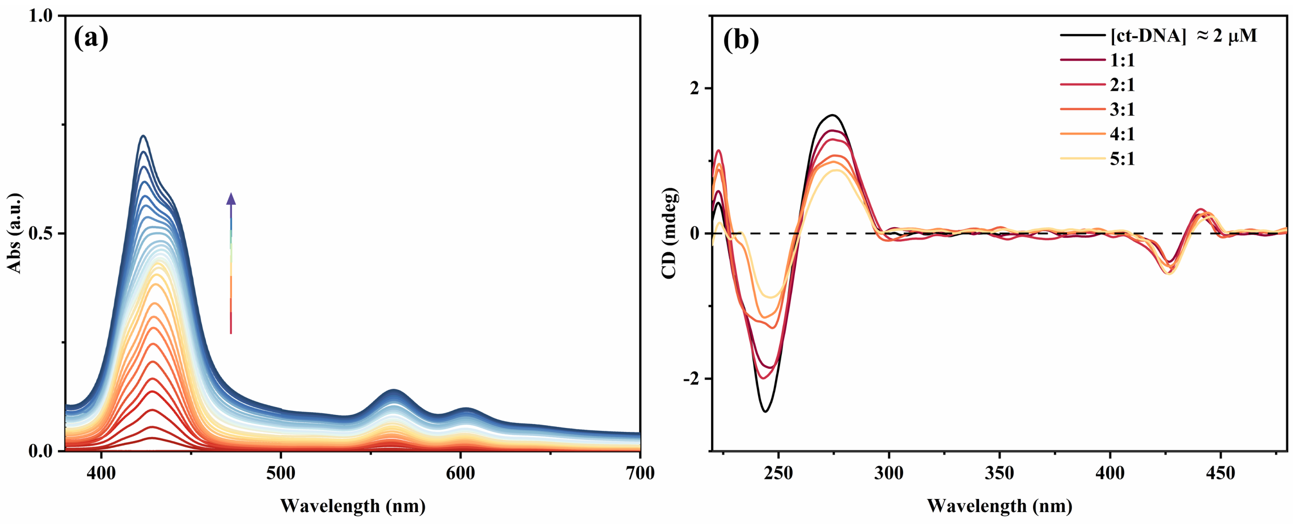

Figure S6. (a) UV–vis titration of ct-DNA (2 µM) with increasing concentrations of ZnTCPPSpm4 (0–14 µM) in 10 mM Tris buffer (pH 7.2) supplemented with 100 mM KCl. ZnTCPPSpm4 was added in 0.5 µM increments. **(b)** CD spectra of ct-DNA (2 µM) in 10 mM Tris buffer (pH 7.2) supplemented with 100 mM KCl in the absence (black line) and in the presence of increasing amounts of ZnTCPPSpm4. Spectra are reported at molar ratios [ZnTCPPSpm4]/[ct-DNA] ranging from 1:1 to 5:1, indicated by a progressive color scale.

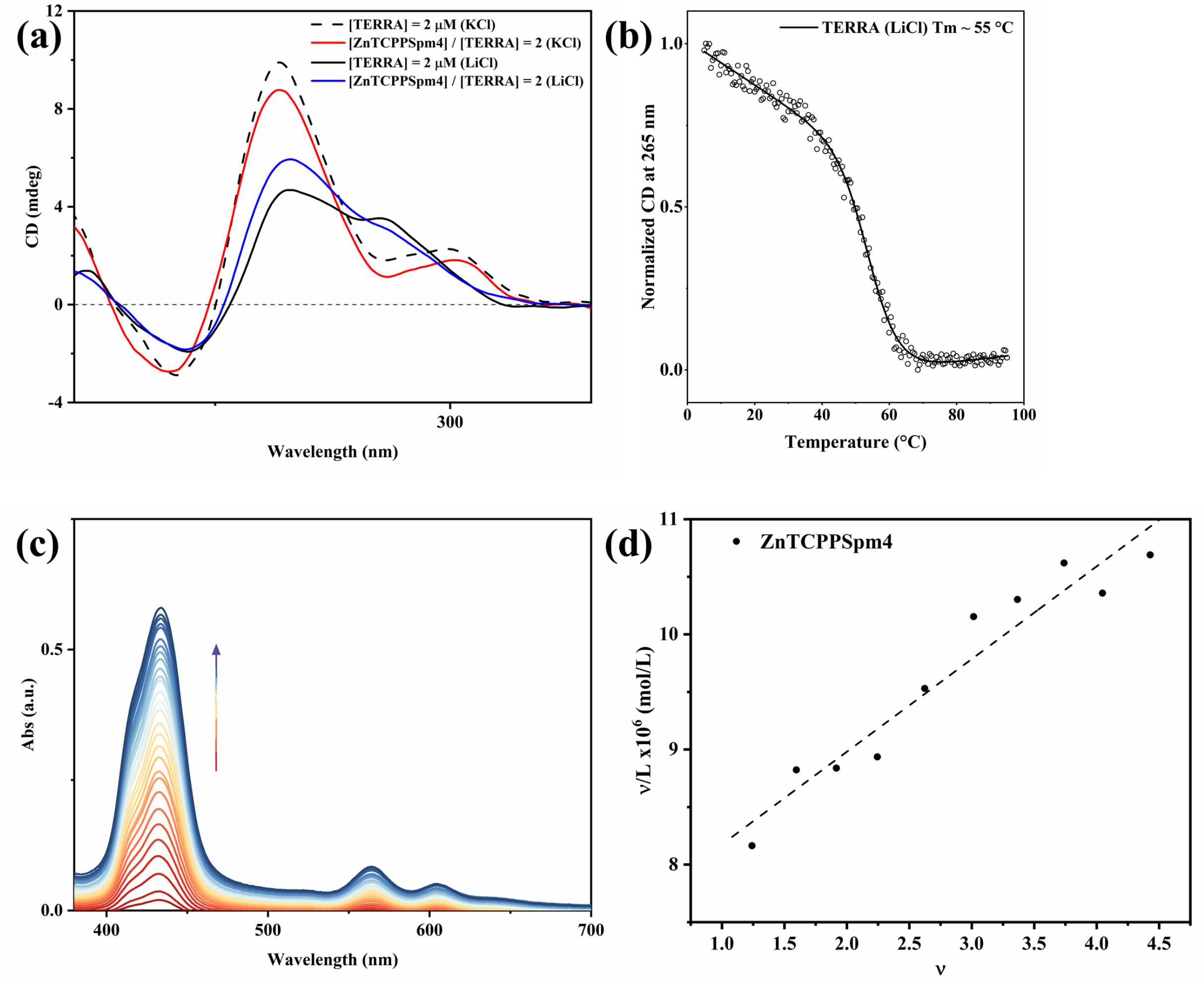

Figure S7. (a) CD spectra of TERRA (2 μM) in 10 mM Tris buffer (pH 7.2) supplemented with 100 mM KCl (black dashed line) and 100 mM LiCl (black line), in the absence and in the presence of ZnTCPPSpm4 at a molar ratio [ZnTCPPSpm4]/[TERRA] = 2 (red and blue lines for KCl and LiCl, respectively). **(b)** CD melting curve of TERRA (2 μM) in 10 mM Tris buffer (pH 7.2) containing 100 mM LiCl, monitored at 265 nm. **(c)** UV–vis titration of TERRA (2 μM) with increasing concentrations of ZnTCPPSpm4 (0–14 μM) in 10 mM Tris buffer (pH 7.2) supplemented with 100 mM LiCl. ZnTCPPSpm4 was added in 0.5 μM increments. **(d)** Scatchard plot obtained from UV–vis titration data for the ZnTCPPSpm4/TERRA system in LiCl, used to determine the apparent binding constant.

References

60. Peacocke, A.R.; Skerrett, J.N.H. The Interaction of Aminoacridines with Nucleic Acids. *Trans. Faraday Soc.* **1956**, *52*, 261–279. https://doi.org/10.1039/TF9565200261.

65. Travagliante, G.; Gaeta, M.; Gangemi, C.M.A.; Alaimo, S.; Ferro, A.; Purrello, R.; D'Urso, A. Interactions between Achiral Porphyrins and a Mature MiRNA. *Nanoscale* **2024**, *16*, 5137–5148. https://doi.org/10.1039/D3NR05504C.
